# COVID-19 Predeparture Test Results and Vaccination Coverage among US-Bound Refugees, 2020–2022

**DOI:** 10.3201/eid3108.250088

**Published:** 2025-08

**Authors:** Ashley S. Judge, Tarissa Mitchell, Alexander Klosovsky, Michelle Weinberg, Christina R. Phares

**Affiliations:** Oak Ridge Institute for Science and Education, Oak Ridge, Tennessee, USA (A.S. Judge); Centers for Disease Control and Prevention, Atlanta, Georgia, USA (A.S. Judge, T. Mitchell, M. Weinberg, C.R. Phares); International Organization for Migration, Washington, DC, USA (A. Klosovsky)

**Keywords:** COVID-19, coronavirus disease, SARS-CoV-2, severe acute respiratory syndrome coronavirus 2, viruses, respiratory infections, vaccination, zoonoses, refugees, United States

## Abstract

We describe predeparture COVID-19 test positivity and vaccination coverage for US-bound refugees. During November 24, 2020–June 11, 2022, a total of 23,972 refugees received 28,465 tests (87% reverse transcription PCR); 2.6% of refugees tested positive. During November 24, 2020–December 31, 2022, vaccination coverage rose from 0% to 71% among 24,831 adult refugees.

The COVID-19 pandemic disrupted global refugee resettlement, an international process offering permanent resettlement to a third country for persons who cannot return to their home country because of persecution on the basis of race, religion, nationality, social group, or political opinion and who can no longer remain in the country in which they reside. The United States suspended resettlement during March 20–July 29, 2020 (*1*,*2*). Upon resumption, the United States mandated pretravel COVID-19 testing for refugees, delaying travel for those who tested positive and their close contacts. During January 26, 2021–June 12, 2022, all travelers, including refugees, were required to show documentation of a negative test or recent recovery from COVID-19 before boarding a US-bound flight (*3*). This requirement was lifted on June 12, 2022 (*4*). US-bound refugees were not required to receive COVID-19 vaccinations but were referred to national programs for vaccination in their country of examination where possible. We assessed the prevalence of positive predeparture COVID-19 tests for all refugees and COVID-19 vaccination coverage for adult refugees. This activity was reviewed by Centers for Disease Control and Prevention’s (CDC)’s National Center for Emerging and Zoonotic Infectious Diseases, Information Collection and Human Studies Team, was deemed not research, and was conducted consistent with applicable federal law and CDC policy (e.g., 45 C.F.R. part 46, 21 C.F.R. part 56; 42 U.S.C. §241(d); 5 U.S.C. §552a; 44 U.S.C. §3501 et seq.).

## The Study

The International Organization for Migration coordinates US travel for refugees and manages many predeparture health assessments. To describe COVID-19 predeparture test results, we analyzed International Organization for Migration testing data for all refugees who entered the United States during November 24, 2020–June 11, 2022 (the last day for which predeparture testing was required for US-bound flights). To determine COVID-19 vaccination coverage, we queried the CDC Electronic Disease Notification system (*5*) for adult refugees (>18 years of age) who entered the United States during November 24, 2020–December 31, 2022. During that period, most COVID-19 vaccinations were administered through national programs; analyses were limited to adults because most countries had not yet extended vaccination programs to minors. Testing and vaccination data were not linked. 

We recorded test type as reverse transcription PCR, rapid antigen test, or other. Refugees could receive >1 test. Persons were considered to have tested positive if >1 test was positive, negative when >1 test was negative and no tests were positive, and unknown when all tests were indeterminate or missing. We calculated the percentage of persons who tested positive by dividing the number of persons with >1 positive result by the number of persons with known results. For the percentage positive over time, we only included persons in calculations for the first period in which they tested positive. We defined completion of a primary vaccine series as receipt of 1 dose of a single-dose vaccine or 2 doses (regardless of time interval) of a 2-dose series authorized in the United States or listed for emergency use by the World Health Organization (WHO). 

Among 24,361 refugees who arrived in the United States during November 2020–June 2022, a total of 29,025 predeparture COVID-19 tests were documented. We excluded 560 missing or indeterminate test results (including results from 389 persons for whom all tests were indeterminate or missing), leaving 23,972 persons receiving 28,465 tests (Appendix). Most (85.0%) persons had 1 test, 12.0% had 2 tests, 2.2% had 3 tests, 0.6% had 4 tests, and 0.2% had 5–10 tests. Among all tests, 2.6% were positive for COVID-19. By type, positivity was 2.7% among 24,853 reverse transcription PCR tests, 2.0% among 3,590 rapid antigen tests, and 9.0% among 22 unspecified tests. Among 23,972 persons, 2.6% had >1 positive test.

**Figure 1:**
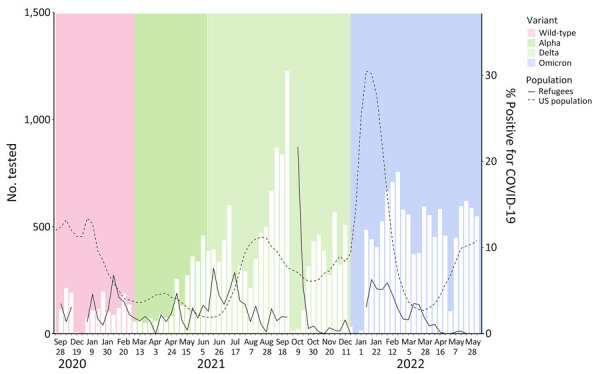
Predeparture COVID-19 test positivity among refugees by week of arrival in the United States (November 24, 2020–June 11, 2022) in study of test results and vaccination coverage among US-bound refugees, 2020–2022. Among refugees, weeks with <20 persons tested were suppressed. White bars indicate number of persons tested; dotted and solid lines indicate percentage positivity. Data for US population COVID-19 test positivity from National Respiratory and Enteric Virus Surveillance System (https://data.cdc.gov/Laboratory-Surveillance/Percent-Positivity-of-COVID-19-Nucleic-Acid-Amplif/gvsb-yw6g/about_data).

The percentage positive was highest in Asia (4.1%), followed by Sub-Saharan Africa (2.9%) and the Americas (2.9%), and lowest in the Middle East/North Africa (1.9%) and Europe (1.8%) (Table 1). Within each region, percentage positive varied by country (Table 1). Over time, the percentage positive ranged from 0% in April 2021 to 21.7% in October 2021 (Figure 1). Across age groups, results were similar, ranging from 2.3% among children <5 years of age to 2.8% among adults >55 years of age.

**Table 1:** Predeparture COVID-19 positivity among US-bound refugees by region and country of overseas medical examination, November 24, 2020–June 11, 2022*

Region and country	No. persons positive	No. persons tested	% Persons with positive test
Total	622	23,972	2.6
Europe	37	2,069	1.8
Moldova	20	312	6.4
Other†	4	201	2.0
Ukraine	13	1,556	0.8
Middle East/North Africa	153	8,228	1.9
Jordan	80	2,922	2.7
Turkey	46	2,032	2.3
Other‡	2	114	1.8
Egypt	24	2,007	1.2
Iraq	1	311	0.3
Lebanon	0	433	0.0
Qatar	0	409	0.0
Sub-Saharan Africa	274	9,318	2.9
Uganda	113	1,177	9.6
Ethiopia	14	397	3.5
Kenya	17	616	2.8
Tanzania	56	2,501	2.2
Zambia	16	725	2.2
Burundi	20	974	2.1
Rwanda	30	1,940	1.5
Malawi	4	362	1.1
Other§	4	626	0.6
Americas	49	1,674	2.9
El Salvador	27	506	5.3
Guatemala	17	702	2.4
Other¶	5	466	1.1
Asia	109	2,683	4.1
Thailand	46	916	5.0
Malaysia	43	911	4.7
Other#	20	856	2.3

*Persons were considered positive if >1 result was positive, negative when >1 result was negative and no tests were positive, and unknown when all results were indeterminate or missing. Number tested excludes persons classified as unknown. Most (88.8%) were tested by reverse transcription PCR, 11.2% by rapid antigen detection, and <0.1% had an unspecified test type. †Albania, Armenia, Austria, Belarus, Germany, Israel, Kazakhstan, Malta, Uzbekistan. ‡Algeria, Bahrain, Kuwait, Oman, Saudi Arabia, United Arab Emirates. §Angola, Botswana, Cameroon, Chad, Côte d'Ivoire, Democratic Republic of the Congo, Djibouti, Ghana, Guinea, Namibia, Niger, South Africa, Sudan, Togo, Zimbabwe. ¶Colombia, Costa Rica, Curaçao, Ecuador, Honduras. #Afghanistan, Australia, Bangladesh, Myanmar, China, Hong Kong, India, Indonesia, Mongolia, Nauru, Nepal, Pakistan, Papua New Guinea, Sri Lanka, Vietnam.

**Figure 2:**
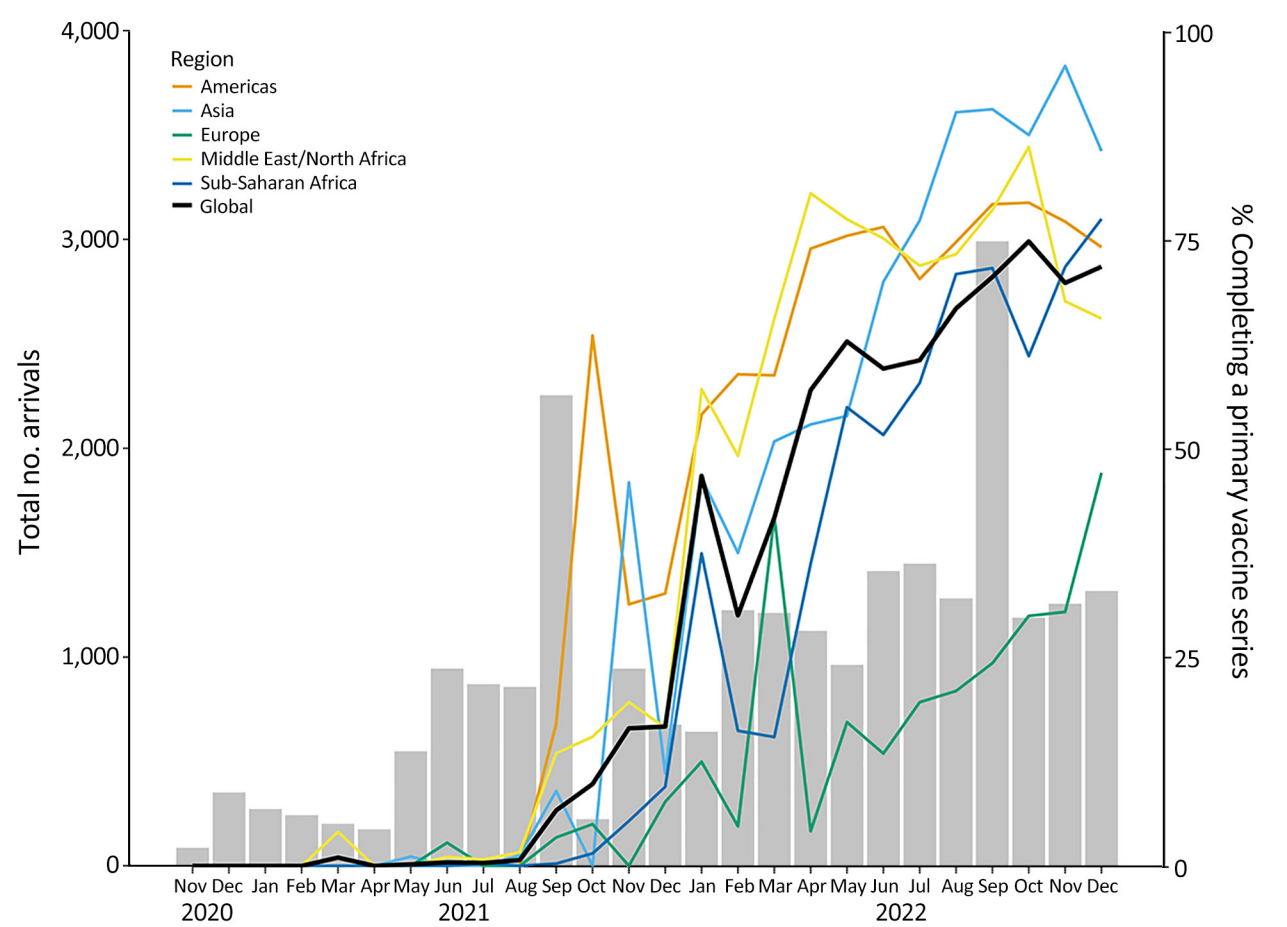
Predeparture completion of a primary COVID-19 vaccine series among adult refugees by month of arrival in the United States (November 24, 2020–December 31, 2022) in study of test results and vaccination coverage among US-bound refugees, 2020–2022. November 2020 represents November 24–30, 2020. Gray bars indicate number of arrivals; colored lines indicate percentage of persons completing primary vaccine series.

During November 2020–December 2022, a total of 24,831 adult refugees arrived in the United States (Appendix). Of those, 2,509 (10.1%) received a single-dose COVID-19 vaccine series, 1,030 (4.1%) received only 1 dose of a 2-dose vaccine series, and 7,878 (31.7%) received 2 doses of a 2-dose vaccine series (Table 2). In total, 41.8% of refugees completed a primary series before arrival in the United States. Coverage increased over time (Figure 2); by December 2022, a total of 71.3% had completed a primary series. The Americas had the highest percentage of persons who completed a primary series (63.4%), followed by Asia (51.0%), Middle East/North Africa (49.4%), Sub-Saharan Africa (34.8%), and Europe (15.7%) (Table 2).

**Table 2:** Predeparture COVID-19 vaccination coverage among adult refugees by region and country of overseas health assessment, November 24, 2020–December 31, 2022

Region and country	No. (%) refugees				Total population
	Single-dose series	Two-dose series		Completed primary series	
		First dose only	First and second dose		
Total	2,509 (10.1)	1,030 (4.1)	7,878 (31.7)	10,387 (41.8)	24,831
Sub-Saharan Africa	2,059 (21.5)	343 (3.6)	1,275 (13.3)	3,334 (34.8)	9,594
Burundi	395 (46.4)	0	0	395 (46.4)	851
Ethiopia	240 (42.7)	9 (1.6)	29 (5.2)	269 (47.9)	562
Kenya	410 (47.6)	10 (1.1)	149 (17.3)	559 (64.9)	862
Malawi	47 (12.3)	8 (2.1)	72 (18.9)	119 (31.2)	381
Rwanda	49 (2.4)	157 (7.5)	742 (35.7)	791 (38.1)	2,079
Tanzania	489 (20.8)	7 (0.3)	17 (0.7)	506 (21.5)	2,352
Uganda	174 (16.4)	112 (10.5)	164 (15.5)	338 (31.9)	1,061
Zambia	94 (12.3)	12 (1.5)	9 (1.2)	103 (13.5)	764
Other*	161 (23.6)	28 (4.1)	93 (13.6)	254 (37.2)	682
Americas	17 (0.7)	239 (10.1)	1,474 (62.7)	1,491 (63.4)	2,352
Ecuador	15 (3.7)	21 (5.2)	299 (74.6)	314 (78.3)	401
El Salvador	0	45 (7.5)	412 (68.8)	412 (68.8)	599
Guatemala	1 (0.1)	134 (13.5)	579 (58.2)	580 (58.3)	994
Honduras	0	23 (7.4)	156 (50.5)	156 (50.5)	309
Other†	1 (2)	16 (32.7)	28 (57.1)	29 (59.1)	49
Asia	16 (0.5)	196 (6.3)	1,579 (50.5)	1,595 (51.0)	3,125
Malaysia	0	107 (7.5)	1,068 (75.3)	1,068 (75.3)	1,419
Thailand	0	63 (7.9)	168 (20.8)	168 (20.8)	806
Other‡	16 (1.8)	26 (2.9)	343 (38.1)	359 (39.9)	900
Europe	63 (2.5)	36 (1.5)	336 (13.2)	399 (15.7)	2,537
Moldova	15 (2.3)	6 (0.9)	78 (11.9)	93 (14.2)	655
Ukraine	1 (0.1)	0	76 (6)	77 (6.1)	1,269
Other§	47 (7.7)	30 (4.9)	182 (29.7)	229 (37.4)	613
Middle East/North Africa	354 (4.9)	216 (3)	3,214 (44.5)	3,568 (49.4)	7,223
Egypt	30 (1.9)	37 (2.3)	264 (16.8)	294 (18.7)	1,573
Jordan	0	77 (3.8)	1,428 (70.4)	1,428 (70.4)	2,027
Lebanon	3 (0.7)	25 (6)	64 (15.5)	67 (16.2)	413
Qatar	273 (30.8)	30 (3.4)	563 (63.5)	836 (94.3)	886
Turkey	0	21 (1.2)	612 (35.8)	612 (35.8)	1,710
Other¶	48 (7.8)	26 (4.2)	283 (46.1)	331 (53.9)	614

*Angola, Botswana, Cameroon, Chad, Côte d’Ivoire, Democratic Republic of the Congo, Djibouti, Ghana, Guinea, Madagascar, Mauritania, Mozambique, Namibia, Niger, South Africa, Sudan, Togo, Zimbabwe. †Colombia, Costa Rica, Curaçao, Peru. ‡Afghanistan, Australia, Bangladesh, Myanmar, China, Hong Kong, India, Indonesia, Mongolia, Nepal, Nauru, Pakistan, Papua New Guinea, Sri Lanka, Vietnam. §Albania, Armenia, Austria, Belarus, Belgium, Czech Republic, Germany, Israel, Italy, Kazakhstan, Kosovo, Kyrgyzstan, Malta, Poland, Russia, Slovak Republic, Slovenia, Tajikistan, Uzbekistan. ¶Algeria, Bahrain, Iraq, Kuwait, Morocco, Oman, Saudi Arabia, United Arab Emirates.

## Conclusions

The COVID-19 pandemic severely disrupted refugee resettlement. Such disruptions can prolong uncertainty and vulnerability for refugees, delay family reunification, and strain resources for host countries and humanitarian agencies (*6*). Even short disruptions can lead to a cascading derailment of administrative processes. Strategies to minimize disruption while preventing pathogen importation were needed.

Universal testing for US-bound refugees represented one such strategy. Despite concerns of heightened infection risk among refugees, predeparture testing showed low prevalence. For 23,972 US-bound refugees, just 2.6% of tests were positive, below the WHO 5% threshold for countries to reopen (*7*) and the 10.1% test positivity found in the general US population during the same period (*8*).

COVID-19 vaccines represented an additional tool to minimize resettlement disruptions while protecting refugees from severe disease; however, vaccine rollout to refugee populations was hampered by variable global distribution of vaccines, resulting in reduced initial access in low- and middle-income countries (*9*). Among US-bound refugees, those who had an overseas health assessment in Middle East/North Africa, Asia, and the Americas had higher COVID-19 vaccination coverage than those assessed in Sub-Saharan Africa or Europe, suggesting greater barriers to vaccination in those regions. In Sub-Saharan Africa, barriers included insufficient vaccine supply and cold chain issues in remote areas. In Eastern Europe, some refugees received vaccines not approved for use in the United States or listed by WHO and thus were not counted in our calculations, and vaccine hesitancy was widespread (*10*). Other challenges included administrative difficulties, requirements for identity documents, and language barriers.

Despite challenges, COVID-19 vaccination coverage among US-bound refugees steadily increased over time, approaching coverage observed for the general US adult population. Within 1 month of US vaccination programs expanding to include adults <65 years of age on April 19, 2021, a total of 57.0% of the general US adult population had received >1 dose (*11*); by December 2022, that figure was 88.4% (*12*). For US-bound adult refugees, 54% had received 1 dose by April 2022 and 71.3% by December 2022, exceeding WHO’s strategic objective for all countries to reach 70% (*13*). Although no vaccinations are mandatory for US-bound refugees, refugees were successfully referred to national vaccination programs in their country of examination for voluntary vaccination.

In conclusion, positivity for COVID-19 among US-bound refugees, which was monitored in real time, remained modest throughout the study period. A predeparture testing strategy further reduced risk by identifying persons who required travel postponement while permitting safe travel for others. Vaccination coverage among US-bound refugees improved over time, drawing near to that of the general US population. Our findings highlight the importance of including refugees in public health initiatives during global health emergencies, demonstrating that such approaches can protect the safety and continuity of resettlement efforts.

Appendix Additional information about COVID-19 predeparture test results and vaccination coverage among US-bound refugees, 2020–2022
