# Supplementary material for: COVID-19 Predeparture Test Results and Vaccination Coverage among US-Bound Refugees, 2020–2022
Source: Emerg Infect Dis. 2025 Aug;31(8):1630–5. doi: 10.3201/eid3108.250088 (PMC12309780; doi:10.3201/eid3108.250088)
Supplement: Appendix — Additional information about COVID-19 predeparture test results and vaccination coverage among US-bound refugees, 2020–2022 [file 25-0088-Techapp-s1.pdf]

Article DOI: https://doi.org/10.3201/eid3108.250088

EID cannot ensure accessibility for supplementary materials supplied by authors. Readers who have difficulty accessing supplementary content should contact the authors for assistance.

COVID-19 Predeparture Test Results and Vaccination Coverage among US-Bound Refugees, 2020–2022

Appendix

Appendix Table. Characteristics of US-bound refugees included in analyses of pre-departure COVID-19 testing (November 24, 2020–June 11, 2022) and vaccination coverage (November 24, 2020–December 31, 2022)

	No. (%)	
Characteristic	COVID-19 testing, N = 23,972*	COVID-19 Vaccination Coverage, N = 24,831†
Age group, y		
0–4	2,009 (8.4)	–
5–11	4,494 (18.7)	–
12–17	3,462 (14.4)	–
18–54	12,493 (52.1)	22,024 (88.7)
≥55	1,514 (6.3)	2,807 (11.3)
Size of Family		
1–3	8,957 (37.4)	21,354 (86.0)
4–6	10,214 (42.6)	3,432 (13.8)
≥7	4,801 (20.0)	45 (0.2)
Region		
Sub-Saharan Africa	9,318 (38.9)	9,594 (38.6)
Americas	1,674 (7.0)	2,352 (9.5)
Asia	2,683 (11.2)	3,125 (12.6)
Europe	2,069 (8.6)	2,537 (10.2)
Middle East/North Africa	8,228 (34.3)	7,223 (29.1)

*Includes all refugees who arrived in the U.S. during November 24, 2020 through June 11, 2022 except 389 individuals for whom all test results were either indeterminate or missing.
†Includes all adult (≥18 y old) refugees who arrived in the U.S. from November 24, 2020 through December 31, 2022.
